# Composite measures of women’s empowerment and their association with maternal mortality in low-income countries

**DOI:** 10.1186/s12884-017-1492-4

**Published:** 2017-11-08

**Authors:** Chiao-Wen Lan, Paula Tavrow

**Affiliations:** Department of Community Health Sciences, UCLA Fielding School of Public Health, 650 Charles E. Young Drive South, Los Angeles, CA USA

**Keywords:** Empowerment, Maternal mortality, Gender equality, Composite measures

## Abstract

**Background:**

Maternal mortality has declined significantly since 1990. While better access to emergency obstetrical care is partially responsible, women’s empowerment might also be a contributing factor. Gender equality composite measures generally include various dimensions of women’s advancement, including educational parity, formal employment, and political participation. In this paper, we compare several composite measures to assess which, if any, are associated with maternal mortality ratios (MMRs) in low-income countries, after controlling for other macro-level and direct determinants.

**Methods:**

Using data from 44 low-income countries (half in Africa), we assessed the correlation of three composite measures – the Gender Gap Index, the Gender Equity Index (GEI), and the Social Institutions and Gender Index (SIGI) – with MMRs. We also examined two recognized contributors to reduce maternal mortality (skilled birth attendance (SBA) and total fertility rate (TFR)) as well as several economic and political variables (such as the Corruption Index) to see which tracked most closely with MMRs. We examined the countries altogether, and disaggregated by region. We then performed multivariate analysis to determine which measures were predictive.

**Results:**

Two gender measures (GEI and SIGI) and GDP per capita were significantly correlated with MMRs for all countries. For African countries, the SIGI, TFR, and Corruption Index were significant, whereas the GEI, SBA, and TFR were significant in non-African countries. After controlling for all measures, SBA emerged as a predictor of log MMR for non-African countries (β = –0.04, *P* = 0.01). However, for African countries, only the Corruption Index was a predictor (β = –0.04, *P* = 0.04). No gender measure was significant.

**Conclusions:**

In African countries, corruption is undermining the quality of maternal care, the availability of critical drugs and equipment, and pregnant women’s motivation to deliver in a hospital setting. Improving gender equality and SBA rates is unlikely to reduce MMR in Africa unless corruption is addressed. In other regions, increasing SBA rates can be expected to lower MMRs.

**Electronic supplementary material:**

The online version of this article (doi:10.1186/s12884-017-1492-4) contains supplementary material, which is available to authorized users.

## Background

Maternal mortality remains a major public health challenge worldwide. It is also considered a social justice issue because women alone suffer from maternal deaths, most of these deaths could be prevented, and women of lower socioeconomic status are at much higher risk [1–3]. Moreover, the global disparity is alarming – almost all maternal deaths (99%) now occur in developing countries, more than half of which occur in sub-Saharan Africa [4]. To address this injustice, the international community agreed that improving maternal health would be one of the eight Millennium Development Goals (MDGs) adopted in 2000. For MDG 5, countries pledged to attain a 75% reduction in their 1990 maternal mortality ratios (MMRs) by 2015. Unfortunately, most countries have failed to reach this target. While the number of maternal deaths worldwide has dropped by 44% since 1990, only approximately 16 countries achieved a 75% reduction by 2015 [4], none of which were in Africa. The gap between the current average MMRs of 239 per 100,000 live births in low-income countries versus 12 for high-income countries sparked a call for the Sustainable Development Goals to set an absolute MMR ceiling of 70 by the year 2030 [5, 6]. This ceiling will be especially difficult for countries in sub-Saharan Africa to reach, as their average MMR is currently at 546 [5].

The causes of the decline in MMR globally since 1990, and its persistence in some countries, are complex and difficult to determine [7]. Interventions targeted at maternal mortality generally focus on increasing women’s access to skilled birth attendance (SBA) and the availability of emergency obstetrical care [8]. However, a recent WHO multi-country survey of maternal and newborn health found that high coverage of essential obstetric care services was not linked to maternal mortality [9]. The study had defined coverage as the proportion of the target population who received the indicated intervention, e.g., the proportion of women with eclampsia who received magnesium sulfate or the proportion of women with sepsis who received a parenteral antibiotic. The researchers concluded that more comprehensive service delivery and early intervention was required. Another study that involved 287,035 inpatients giving birth in health facilities in 24 low- and middle-income countries found that women with 6 years or less of education had twice the risk of maternal mortality than those with more than 12 years of education [10], suggesting that holistic approaches, which include raising women’s status, might be more effective in reducing MMRs.

In the past few decades, many changes have occurred in women’s lives that may be affecting MMRs through the mechanism of female empowerment. By empowerment, we mean increasing women’s agency and their ability to make strategic life choices [11]. Among the most noteworthy changes occurring globally that have contributed to heightened women’s empowerment are rising female educational levels [12], later ages of marriage [13], more women in the formal labor force [14], and more women in political positions such as Parliament [15]. These changes have elevated women’s status and earning potential, such that their social worth is less dependent on the number of children they produce. This, in turn, has paved the way for women’s greater use of contraceptive methods, which have enabled women to have more control over their family size. Researchers have found a strong correlation between total fertility rates and MMRs – when women are able to limit their absolute number of births, they reduce the number of times they are exposed to the risk of mortality, as well as their likelihood of having a life-threatening pregnancy due to high parity [16, 17]; both of these factors could make total fertility rate (TFR) a predictor of MMR. In addition, more empowered women (based on education, wealth, and autonomy) are more likely to use contraception, attend antenatal clinics, and utilize SBA – all factors that could contribute to lower maternal deaths [18, 19].

Another mechanism by which female empowerment could affect MMRs is in the political arena. When women have more political power at the national or regional level, they may try to ensure that services which have been neglected, such as maternity care or access to safe abortion, receive more funding and attention [20]. In seeking to understand the striking disparities in MMRs, some researchers have recognized the importance of political will [2, 8]. A case study analysis of five low-income countries concluded that international funding and technical interventions were not sufficient to achieve reductions in maternal mortality, since countries have many competing priorities [2]. Instead, democratic transitions and political leadership were strong influences on whether maternal health received adequate attention. Other analysts have similarly determined that political commitment is essential [21, 22]. Interestingly, none of these studies specifically analyzed whether having more women in political leadership roles had a bearing on maternal mortality. However, evidence suggests that female policymakers are more inclined to prioritize health and social welfare programming, including maternal healthcare [23, 24]. For instance, a study in India found that, for every one standard deviation increase in the number of female political representatives, a 1.5% reduction in neonatal mortality occurred, because women politicians were more likely to support antenatal care and health facilities [25]. Similarly, in Rwanda, the rise of women parliamentarians led to a major increase in national budget expenditures on healthcare, from 3% in 1998 to 12% in 2006, directly attributed to women parliamentarians’ lobbying activities [26]. After countries introduce gender quotas that increase political participation by women, they tend to spend 3.4 percentage points more on social welfare than countries without them [24].

The persistently high rates of MMR in most of sub-Saharan Africa still are not well understood [27]. A provocative article by Mostert et al. [28] posits that corruption in healthcare systems in African countries is hindering the quality and accessibility of care to patients suffering from cancer, thereby contributing to lower cancer survival rates. The authors noted that corruption could lead to resource misallocation, procurement of inferior equipment and drugs, theft and resale of essential products, preferential treatment to those who can afford care, and bribes for services. All of these affect the overall quality of health services, which could mean that corruption is also having an impact on MMRs in Africa. A study focused on sub-Saharan African countries found that three groups of variables, related to female education, national income, and an effective health system, all seemed to be associated with MMR [1], with none more significant than the others. However, the authors did not include corruption as a variable, and it is therefore not known if it was influential.

To date, several ecological studies have sought to assess whether measures of human development could explain national MMRs. Two studies examined the Human Development Index and found that it was linked to MMR [29, 30], but they did not assess whether women’s empowerment was a factor. Another two studies specifically used a composite indicator of women’s empowerment [31, 32] to assess if greater gender equity was linked to lower MMR. In the Varkey et al. study [32], the authors employed the Gender Empowerment Measure (GEM) to examine the correlations between women’s empowerment and a range of health indicators, including maternal mortality, for the 75 countries in the 2006 Human Development Report. The GEM was developed by the United Nations Development Program (UNDP) to measure three dimensions of female empowerment, namely economic participation and decision-making, political participation, and power over economic resources. After adjusting for gross domestic product (GDP) per capita, Varkey et al. [32] found that the GEM was not correlated with maternal mortality, although it was associated with several other health indicators (low birth rate, fertility rate, infant mortality rate, and child mortality rate). In the Choe et al. study [31], the authors used the Gender Gap Index (GGI) to assess the effects of gender equality on MMRs in 134 countries using 2010 data. The GGI was developed by the World Economic Forum and has four dimensions, namely education, economic, political, and health. After controlling for several economic and health systems variables, the authors found that the GGI was not significantly correlated with MMR.

Given the unexpected results of the Varkey et al. [32] and Choe et al. [31] studies, we wondered whether their conclusions that there were no macro-level associations between female empowerment and MMR could be related to methodological or measurement issues. We felt that their studies raised some critical questions. First, since various indices of gender equality exist, none of which has been universally adopted [33], might another measure be more closely associated to MMR than the ones previously tested? Second, could it be that gender equality has a lagged effect; in other words, could women’s empowerment –particularly in the political arena – translate into lower MMRs later, rather than immediately? Third, might there be regional differences in how female empowerment affects MMRs, which are masked when all low-income countries are examined together? Finally, given the political nature of maternal mortality, could transparency and democratization be important additional factors in accounting for MMRs?

To answer these questions, we designed a study that would (1) compare the association with MMR of three of the most widely-accepted composite measures of women’s empowerment; (2) examine the correlations with a contemporaneous measure of MMR as well as a lagged measure; (3) compare African countries as a whole to an equivalent set of other low-income countries, to determine if other factors could account for the persistently low MMR in the sub-Saharan African region; and (4) investigate some measures of political accountability, such as corruption, which have been missing from previous studies. Our expectation was that this approach would enable us to revisit the issue of whether a composite measure of female empowerment is associated with MMR after controlling for other factors, and possibly arrive at a different conclusion.

## Methods

We employed an ecological study design to assess the association between female empowerment indices, GDP per capita, political indicators, health service indicators, and MMRs among the low-income countries. Each country served as a unit of analysis. The selected low-income countries were those indicated as such by the World Bank. Countries with missing data from the chosen variables were excluded. This resulted in a total of 44 countries included in the study, consisting of 22 sub-Saharan African countries and 22 non-African countries. The sub-Saharan African countries were Benin, Burkina Faso, Cameroon, Chad, Cote d'Ivoire, Ethiopia, Gambia, Ghana, Kenya, Lesotho, Madagascar, Malawi, Mali, Mauritania, Morocco, Mozambique, Nigeria, Senegal, Tanzania, Uganda, Zambia, and Zimbabwe. The non-African countries were Armenia, Bangladesh, Bolivia, Cambodia, Egypt, El Salvador, Georgia, Guatemala, Guyana, Honduras, India, Indonesia, Kyrgyz Republic, Moldova, Nicaragua, Pakistan, Philippines, Sri Lanka, Tajikistan, Ukraine, Vietnam, and Yemen. All national data were drawn from the 2016 annual report of the World Bank [34], unless specified otherwise.

### Measures of women’s empowerment

A number of indices of female empowerment have been developed that aggregate multiple indicators from different dimensions and provide a national score (Table 1). A recent study assessed the reliability and validity of five gender indices that are publicly available to researchers and policymakers [33]. The measures assessed were the UNDP’s GEM, the UNDP’s Gender-related Development Index (GDI), Social Watch’s Gender Equity Index (GEI), the World Economic Forum’s GGI, and the OECD’s Social Institutions and Gender Index (SIGI). Of these, the GEM and GDI were phased out by UNDP in 2009 and replaced with the Gender Inequality Index (GII); however, because the GII includes maternal mortality as one of its indicators of female empowerment, it cannot be used for our purposes. This leaves three indices (GGI, GEI, and SIGI) available for this study. For all of the gender measures, we used the 2012 scores. According to Hawken and Munck [33], each of these three indices has both strengths and weaknesses, and none is recommended over another:

- The GGI examines the gap, or inequality, between men and women in four categories: (1) economic participation and opportunity – using data on salaries, participation levels, and access to high-skilled employment; (2) educational attainment – using data on access to basic and higher levels of education; (3) political empowerment – using data on representation in decision-making structures; and (4) health and survival – using data on life expectancy and sex ratio at birth. The GGI aggregates 14 indicators that are intended to compare how evenly countries are allocating their resources and opportunities among their male and female populations. The data is then scaled from 0 to 1, with 1 being no inequality [35].
- The GEI computes a value for the gender gap in each of the three areas: (1) economic participation – using data on gaps in income and employment; (2) educational attainment – using data on enrollment at all levels and literacy; and (3) empowerment – using data on gaps in highly qualified jobs, parliament, and senior executive positions. Each item is scaled from 0 (when, for example, no woman is educated and all men are) to 100 (perfect equality). The GEI score is an average of these three dimensions, from 0 to 100, with 100 being no inequality [36].
- The SIGI is a measure of discrimination against women in social institutions (formal and informal laws, social norms, and practices). It covers five dimensions of social institutions, spanning major socioeconomic areas that affect women’s lives: discriminatory family code, restricted physical integrity, son bias, restricted resources and assets, and restricted civil liberties. The SIGI’s variables quantify discriminatory social institutions such as unequal inheritance rights, early marriage, violence against women, and unequal land and property rights. The measure is scored from 0 to 1, with 0 being the optimum. Unlike the previous two measures, a lower score indicates less discrimination against women [37].

**Table 1:** Gender indices, components, and use in this study

Gender index	Abbreviation	Years data available	Source	Main components	Scale	Countries in 2012	Used in this study
Gender Empowerment Measure	GEM	1995–2009	UNDP	1) portion of seats held by women in national parliaments; 2) percentage of women in economic decision-making positions; 3) female share of income	0–1, equality =1	–	
Gender-related Development Index	GDI	1995–2009	UNDP	1) life expectancy; 2) educational attainment; 3) adjusted real income – after taking note of inequalities between women and men	0–1, equality = 1	–	
The Global Gender Gap Index	GGI	2006–present	World Economic Forum	1) economic participation and opportunity; 2) educational attainment; 3) political empowerment; 4) health and survival	0–1, equality = 1	135	√
Gender Equity Index	GEI	2007, 2008, 2009, 2012	Social Watch	1) education; 2) economy; 3) political empowerment	0–100, equality = 100	168	√
Social Institutions and Gender Index	SIGI	2009, 2012, 2014	OECD	1) discriminatory family code; 2) restricted physical integrity; 3) son bias; 4) restricted resources and entitlement; 5) restricted civil liberties	0–1, equality = 0	88	√
Gender Inequality Index^a^	GII	2010, 2011, 2014	UNDP	1) reproductive health; 2) empowerment (political and higher education); 3) labor market participation	0–1, equality = 0	148	

*UNDP* United Nations Development Program, *OECD* Organization for Economic Cooperation for Development

^a^GII was a new measure introduced in 2010 aimed to ameliorate some of the problems associated with the GEM and GDI

### Other measures

The outcome of interest for this study was the MMR, which is the number of deaths among women from any cause related to or aggravated by pregnancy or its management (excluding accidental or incidental causes) during pregnancy, childbirth, or within 42 days of termination of pregnancy, irrespective of the duration or site of the pregnancy, for every 100,000 live births in a given year or period of time [38]. MMR data were obtained from the World Bank [34]. We chose to test both MMR 2012 and MMR 2015 to assess if there appeared to be lag effect of women’s empowerment on maternal mortality. Additionally, we included MMR in 1990 to calculate which countries met the MDG 5 goal of 75% reduction in MMR from 1990 to 2015. However, among the countries included in this study, only one country (Cambodia) achieved the MDG 5, thus preventing us from performing sub-analyses comparing the countries that met MDG 5 and those that did not.

For our analyses, we included two health-related indicators widely held to be linked to MMRs. SBA is the proportion of births attended by skilled health personnel, calculated by the proportion of total live births that are attended by skilled birth personnel trained in providing lifesaving obstetric care [39]. A limitation of this variable is that the proportion is not collected routinely, so we used the most recent figure available (from 2007–2014). We also included TFR, which is an accepted fertility measure, calculated by the number of children who would be born per woman if she were to pass through her reproductive years bearing children according to the current schedule of age-specific fertility rates [40].

The remaining measures included in this study were one economic variable and two separate composite political indices of transparency and accountability. The GDP per capita is a measure of total output of a country that takes the GDP and divides it by the number of people in the country. We chose to use per capita GDP because it shows the relative performance of countries and is commonly used to estimate economic well-being [41]. GDP was used to estimate well-being rather than the Human Development Index, because the HDI incorporates educational parity and would therefore overlap with the gender composite measures under review. For political measures, we relied on two indices that have been recognized globally as measures of political accountability:

- The Corruption Perceptions Index ranks countries and territories based on how corrupt a country’s public sector is perceived to be. It is a composite index, a combination of opinion surveys and expert assessments of corruption, collected by a variety of institutions specializing in governance and business climate analysis. Transparency International reviews the methodology of each data source and has been publishing the Corruption Perceptions Index annually since 1995 as an indicator of administrative and political corruption. Corruption is defined as an “*abuse of entrusted power for private gain*” [42]. The index encompasses bribery, extortion, and nepotism. A country or territory’s score indicates the perceived level of public sector corruption on a scale of 0 to 100, where 0 means that a country is perceived as highly corrupt and 100 means it is perceived as very clean. Although it focuses on perceptions of corruption rather than the actual extent of corruption, the index has been assessed to be a reliable and consistent measure [43]. We used the 2012 scores because they were available for all countries in our review.
- The Freedom in the World survey, conducted by the human rights organization Freedom House, provides an annual evaluation of the state of global freedom as experienced by individuals [44]. The Freedom House Index consists of political rights, civil liberties, and freedom. Political rights include the right to vote freely for distinct alternatives in legitimate elections, compete for public office, and join political parties and organizations. Civil liberties consist of associational and organizational rights, and the rule of law. Freedom encompasses freedom of expression and belief, as well as personal autonomy without interference from the state. The elements of the survey were derived from the Universal Declaration of Human Rights. Each country and territory is assigned a numerical rating, on a scale of 1 to 7, for each of the three categories, with 1 indicating the highest degree of freedom and 7 the lowest. Total scores range from 3 to 21. For this study, we recalculated the composite score so that higher scores indicate more freedom and rights within a country in 2012.

### Data analysis

All statistical analyses were performed using SPSS (Statistical Package for Social Sciences) software, version 23. First, we calculated means and standard deviations of the various measures, disaggregated by region. Second, we estimated Pearson correlation coefficients to examine the relationship between the various macro-level measures and the MMR. We tested the three composite measures of women’s empowerment (GEI, GGI, and SIGI), SBA, and TFR, as well as economic (GDP per capita) and political (Corruption Index, Freedom House Index) measures from 2012 to assess which measure seems most closely associated with MMRs in two time intervals, namely 2012 and 2015 (to examine lag effects). Sub-analyses were also performed to determine if there were regional differences between sub-Saharan African and non-African countries. Third, we conducted a series of multiple regression analyses to identify which variables were significant after controlling for the others. We report the regression coefficients with their significance levels, as well the extent of variance explained by each model.

For the regression analyses, we assessed normality of residuals and appropriateness of other regression assumptions using standard plots and tests of normality [45]. When necessary, the indicator variables were transformed to meet regression assumptions [46]. A logarithmic transformation of MMR and GDP per capita were deemed appropriate. The scale of GEI was made comparable to the other two gender indices (SIGI and GGI) by dividing the original score by 100, so that all would be on the 0–1 scale.

## Results

A summary of the mean values of the indicators for the countries included in the study is provided in Table 2. Sub-Saharan African countries, in comparison to non-African countries, had significantly higher MMRs in 2012 and 2015 (518 vs. 125 in 2012; 480 vs. 113 in 2015). Among the three gender indices, SIGI was significantly worse in African countries compared to non-African countries (*P* = 0.02), while there was no difference in GEI and GGI. In addition, we found a statistically significant difference in GDP per capita between the two groups, with average GDP per capita in sub-Saharan African countries being approximately 50% less than the other countries. SBA and TFR also differed by region, with sub-Saharan African countries having much lower SBA and higher TFR. However, there were no significant differences by region in either of the political indicators of civil liberties or corruption.

**Table 2:** Maternal mortality, women’s empowerment, economic, political, and health measures by region

	Sub-Saharan African countries (n = 22)		Non-African countries (n = 22)		Total (n = 44)		*P* value
	Mean	SD	Mean	SD	Mean	SD	
*Maternal mortality ratio*							
MMR 2012 MMR 2015	518 480	193 185	126 114	98 90	322 297	250 235	0.00 0.00
*Gender empowerment measures*							
GEI 2012^a^ GGI 2012^a^ SIGI 2012^b^	0.51 0.65 0.32	0.13 0.05 0.12	0.59 0.67 0.24	0.15 0.06 0.11	0.55 0.66 0.28	0.14 0.06 0.12	0.07 0.44 0.02
*Economic*							
GDP per capita 2012^c^	2485	1467	5881	2428	4183	2623	0.00
*Political*							
Freedom House Index 2012^a^ Corruption Index 2012^a^	8.4 32.6	4.3 6.6	8.9 31.2	3.6 7.0	8.6 31.9	3.9 6.7	0.69 0.49
*Health*							
Skilled Birth Attendance 2007–2014^d^ Total Fertility Rate 2015	59.0 4.9	17.4 0.9	82.6 2.6	19.2 0.8	70.8 3.7	21.7 1.4	0.00 0.00

*MMR* maternal mortality ratio, *GEI* Gender Equity Index, *GGI* Global Gap Index, *SIGI* Social Institutions and Gender Index, *SD* standard deviation.

^a^Higher score indicates more equality, less perceived corruption or more freedom

^b^Lower score indicates more equality

^c^GDP per capita is in USD

^d^Using most recent survey available for each country, percent of births attended by skilled provider

The correlation coefficients among MMR, gender empowerment measures, and the other indicators for the entire sample of low-income countries are shown in Table 3. In comparing the three gender indices, we found that both GEI and SIGI were significantly correlated with MMR (2012 and 2015), but not GGI. GDP per capita was also significantly correlated with MMR in both of the time intervals, as well as SBA and TFR, but not with any other indicator. While neither of the political indices was associated with MMR, the Freedom House measure did correlate with two of the gender indices (GEI and GGI). While all three gender indices were strongly correlated, GEI and GGI had the highest correlation (0.89). Not surprisingly, the Freedom House Index was highly correlated with Corruption Index (0.57). The MMRs for 2012 and 2015 were so strongly correlated (0.99) as to suggest that they have not been independently calculated.

**Table 3:** Correlation coefficients and significance levels among selected variables in all low-income countries (n = 44)

	1	2	3	4	5	6	7	8	9
1. MMR 2012									
2. MMR 2015	0.99**								
3. GEI 2012	–0.43**	–0.43**							
4. GGI 2012	–0.25	–0.24	0.89**						
5. SIGI 2012	0.54**	0.55**	–0.64**	–0.55**					
6. GDP per cap. 2012	–0.64**	–0.63**	0.22	0.01	–0.30				
7. Freedom House 2012	0.20	0.20	–0.28	–0.37*	0.16	–0.17			
8. Corruption Index 2012	–0.17	–0.18	0.27	0.26	–0.05	0.23	–0.56**		
9. Skilled birth attendance	–0.67**	–0.63**	0.58**	0.42**	–0.52**	0.53**	–0.20	0.22	
10. Total fertility rate 2015	0.83**	0.83**	–0.48**	–0.29	0.56**	–0.68**	0.14	–0.14	0.67**

*MMR* maternal mortality ratio, *GEI* Gender Equity Index, *GGI* Gender Gap Index, *SIGI* Social Institutions and Gender Index

*P < 0.05; ** *P* < 0.001

Some of these correlations changed when examined by region. The correlation coefficients among MMR, gender empowerment measures, and the other indicators for the sub-Saharan African countries are shown in Table 4. Compared to when all the low-income countries were present, for the sub-Saharan African countries, no significant correlation was observed between MMR and GDP per capita or SBA, which was unexpected. With regard to the gender indices, only the SIGI was correlated with MMR (in both time intervals). Of the political indicators, MMR was found to be significantly correlated with the Corruption Index. The correlations for the non-African countries are shown in Table 5. Interestingly, for these countries, only the GEI was correlated to MMR. None of the other economic or political indicators were associated with MMR, while both SBA and TFR were highly correlated.

**Table 4:** Correlation coefficients and significance levels among selected variables in sub-Saharan African countries only (n = 22)

	1	2	3	4	5	6	7	8	9
1. MMR 2012									
2. MMR 2015	0.98**								
3. GEI 2012	–0.30	–0.30							
4. GGI 2012	–0.21	–0.21	0.91**						
5. SIGI 2012	0.49*	0.50*	–0.53*	–0.44					
6. GDP per cap. 2012	–0.20	–0.18	–0.19	–0.38	–0.23				
7. Freedom House 2012	–0.38	0.35	–0.29	–0.37	0.03	–0.05			
8. Corruption Index 2012	–0.48*	–0.50*	0.37	0.33	–0.30	0.10	–0.69**		
9. Skilled birth attendance	0.36	–0.27	0.28	0.31	0.28	0.09	–0.39	0.38	
10. Total fertility rate 2015	0.52*	0.51*	–0.26	–0.16	0.69**	–0.43*	0.02	–0.31	–0.40

*MMR* maternal mortality ratio, *GEI* Gender Equity Index, *GGI* Gender Gap Index, *SIGI* Social Institutions and Gender Index

*P < 0.05; ** *P* < 0.001

**Table 5:** Correlation coefficients and significance levels among selected variables in non-African countries only (n = 22)

	1	2	3	4	5	6	7	8	9
1. MMR 2012									
2. MMR 2015	0.99**								
3. GEI 2012	–0.54**	–0.54*							
4. GGI 2012	–0.38	–0.38	0.89**						
5. SIGI 2012	0.40	0.40	–0.67**	–0.63**					
6. GDP per cap. 2012	–0.40	–0.40	0.15	0.06	–0.01				
7. Freedom House 2012	–0.02	< 0.01	–0.26	–0.38	0.33	–0.29			
8. Corruption Index 2012	–0.40	–0.40	0.28	0.23	0.06	0.59**	–0.44*		
9. Skilled birth attendance	–0.71**	–0.68**	0.73**	0.52*	–0.54*	0.40	0.02	0.30	
10. Total fertility rate 2015	0.56**	0.57**	–0.66**	–0.51*	0.30	–0.29	0.36	–0.49*	–0.54**

*MMR* maternal mortality ratio, *GEI* Gender Equity Index, *GGI* Gender Gap Index, *SIGI* Social Institutions and Gender Index

*P < 0.05; ** *P* < 0.001

Based on the correlation results, we developed multiple regression models of log MMR 2012 and 2015 using all of the variables that had previously been found associated with MMR in bivariate correlations, be they with all low-income countries, sub-Saharan African countries only or non-African countries only (Table 6). We only show the regression models with MMR 2015, because MMR 2012 had the same outcomes. When examining the countries as a whole, a higher proportion of SBA and lower TFR were associated with lower levels of MMR (β = –0.02, *P* = 0.02; β = 0.41, *P* < 0.01, respectively) after controlling for the other variables. This means that, if women overall had one less child, MMR would decrease by 41%. Additionally, for every 1% increase in SBA overall, MMR would decrease by 2%. The model explained 75% of the variance in MMR. None of the gender composite measures were significant. When examining sub-Saharan African countries, none of the measures were significant expect for the Corruption Perceptions Index (β = –0.04, *P* = 0.04), which suggests that a lower perceived corruption predicted lower MMR. Among non-African countries, only SBA remained significant after adjusting for other variables in the model (β = –0.04, *P* = 0.01). This indicates that increasing SBA rates in non-African countries would be influential in lowering MMR, with every 1% increase in SBA likely to decrease MMR by 4%.

**Table 6:** Multiple regression of logarithm maternal mortality ratio (2015) on empowerment measures, GDP per capita, Corruption Index, skilled birth attendance, and total fertility rate by region

	Sub-Saharan African countries (n = 22)		Non-African countries (n = 22)		Total (n = 44)	
	β	*P* value	β	*P* value	β	*P* value
GEI 2012	–0.08	0.94	0.76	0.68	–0.73	0.45
SIGI 2012	0.59	0.65	–1.96	0.31	–1.26	0.25
GDP per cap. 2012^a^	–0.28	0.20	–0.14	0.74	–0.29	0.21
Corruption Index 2012	–0.04	0.04	0.01	0.82	0.01	0.72
Skilled birth attendance	0.01	0.31	–0.04	0.01	–0.02	0.02
Total fertility rate 2015	0.13	0.45	0.32	0.23	0.41	< 0.01
R^2^	0.43		0.51		0.75	

*β* Standardized beta coefficient, R^2^ adjusted R square

^a^GDP per capita was log transformed to meet regression assumptions

## Discussion

There is a burgeoning literature suggesting that women’s health is affected by their unequal status in society, whether in the form of limited access to educational and economic opportunities, through gender discrimination in society, or through lack of voice and agency in public and private settings [47]. The findings of the current study suggest that, for the 44 low-income countries included in the study, higher female empowerment as measured by gender composite indices was not associated with lower MMRs after controlling for other measures. This finding held when the countries were disaggregated by region. However, a surprising result was that SBA was only predictive of MMR in non-African countries. For sub-Saharan African countries, perceived corruption as measured by the Corruption Index was the only significant predictor of MMR.

Our results replicate the previous finding by Choe et al. [31] that GGI was not a significant predictor of maternal mortality after controlling for GDP per capita [31]. However, our results also suggest that GGI had less relationship to MMR than the other two gender indices when analyzing low-income countries from multiple regions. Even though the three measures had overlapping components and showed significant correlations among themselves, each measure had a unique focus that may have affected its association with MMRs. GGI included health and life expectancy components which may have diluted the “ability to make strategic life choices” (i.e., empowerment) [11] aspect of the measure. SIGI focused on five areas in which women and girls faced discrimination, but discrimination may not be the main driver of MMRs, as only women can die in childbirth. Moreover, as Hawken et al. [33] noted, SIGI may be “a measure that holds much promise but has been poorly executed” due to lack of adequate data from most countries. The combination of educational parity, economic opportunity, and political power as measured by the GEI may encapsulate the dimensions of female empowerment most likely to enable women – in the private and public spheres – to control their fertility but, like SIGI, it was not linked to MMR when other variables were controlled.

Our attempt to determine if there was a lag effect of female empowerment on maternal mortality was weakened by limitations of the national MMR data available to researchers. The exceedingly high level of correlation between the MMRs of 2012 and 2015 indicates that annual MMRs are being mathematically calculated and hence may be subject to considerable error. It is possible that, if a longer lag interval were used, such as 5 years or more, countries would have had time to conduct new MMR surveys that would have yielded more divergent MMRs. Our dilemma was that our first priority was to compare recent gender indices, so we were not able to structure the study to have a lag time longer than 3 years between MMRs.

In seeking to assess if regional differences existed between factors associated with MMRs, we found that none of the gender indices studied was robust enough to continue to be significant when adjusted for other variables. In contrast, for countries in sub-Saharan Africa, the Corruption Index was correlated with MMRs in bivariate analysis and remained significant in multiple regression analysis. We believe that this was a noteworthy finding. The Corruption Index is a measure of national governance and a proxy for effective health system stewardship. Even though perceived corruption in the sub-Saharan African region was not significantly higher than in non-African countries, only in sub-Saharan Africa was it strongly associated with MMR. The more corrupt a government was perceived to be (i.e., the lower Corruption Perception Index score), the higher its maternal mortality.

Corruption is a highly complex and multifaceted obstacle for development across the globe [48]. There is mounting evidence of the negative effects of corruption on population health [49–51]. Indeed, the lack of good governance and accountability, coupled with corruption, has been called one of the biggest challenges in improving health services delivery in the African continent [52, 53]. Although political leaders from African countries had vowed to spend at least 15% of their annual budget to improve the health sector by 2015 in the Abuja Declaration, almost no African countries accomplished this Millennium Development Goal [54]. Health workers in sub-Saharan Africa have affirmed that primary care is highly susceptible to corruption [55]. Studies from several African countries, including Uganda, Tanzania, and Angola, have documented how unauthorized user fees/informal payments during pregnancy and childbirth deter poor women, who are most susceptible to maternal morbidity and mortality, from giving birth in health facilities [56, 57]. Moreover, other forms of corruption in the health sector, including hospital detention practices and biased fee waiver procedures, hamper efforts to improve population health in African countries [25].

Although traditional anti-corruption initiatives have employed a gender-neutral approach, several civil society organizations are calling for women to take a more active role in addressing health sector corruption, because corruption appears to disproportionately affect them [48]. One strategy is to increase women’s participation in healthcare governance and in monitoring service delivery. Another strategy is to incorporate women’s organizations, such as associations of women journalists, into mobilizing public action against corrupt practices. For example, women’s organizations in the Philippines have been at the forefront of anti-corruption efforts, including exposing a high-level criminal network that was trafficking in women and misusing public funds [58]. In general, women seem more sensitive to corruption and less corruptible when they are in positions of power [58]. Thus, efforts to achieve gender equality in the political and management spheres are also considered anti-corruption strategies. Further research is needed to better quantify and diagnose corruption and other problems in basic service provision in developing countries. It may be beneficial to augment the Corruption Index with household or patient surveys to capture the experience-based indicator of corruption within the country [59].

This study had several limitations. First, all ecological studies have a potential limitation of ecological fallacy; that is, associations observed at an aggregated level do not necessarily represent associations that exist at an individual level [60]. Second, this study was a cross-sectional analysis at the country level and therefore we cannot draw causal inferences from the results. In addition, the outcome is estimated using a large uncertainty interval. As we have used countries as the unit of analysis, we cannot provide any information about variation within each nation. Healthcare infrastructure and the economic environment may vary throughout a country, and these differences could be masked by the country-level data. Third, our selection of indicators and measures was constrained by data availability by country, and therefore does not include a complete list of all low-income countries. In addition, the gender indices and other measures used in this study rely on data of differing levels of accuracy, which cannot be independently assessed.

## Conclusions

Composite measures of female empowerment may be valuable tools in helping to set the development agenda and in advancing women’s health and rights. However, in comparing different gender indices’ associations with MMRs in low-income countries, this study found that none of them were robust. This suggests that either the gender indices themselves require some re-calibration or that female empowerment’s effects on maternal mortality are not as significant as has been theorized. Future studies are needed to determine if these gender indices are associated with other facets of women’s health such as maternal morbidities, depression, and chronic conditions. If none emerge as consistently predictive, it may be necessary to revisit how they are constructed and to consider alternate elements.

This study’s key finding is that corruption, while not different in scale between sub-Saharan African and non-African countries, is discouraging African women from delivering in health facilities and reducing the quality of maternal care offered. Unless concerted efforts to reduce or eliminate corruption in healthcare systems in sub-Saharan African countries are undertaken, including elimination of unauthorized user fees [61], auditing of healthcare expenditures, holding service providers accountable for quality of care in public institutions, and greater civil society involvement in health governance, women in African countries will continue to suffer disproportionately higher MMRs.

## Additional file

Additional file 1: Open peer review. (PDF 83 kb)

## Abbreviations

GDI: Gender-related Development Index
GEI: Gender Equity Index
GEM: Gender Empowerment Measure
GGI: Gender Gap Index
GII: Gender Inequality Index
MDGs: Millennium Development Goals
MMRs: maternal mortality ratios
SBA: skilled birth attendance
SIGI: Social Institutions and Gender Index
TFR: total fertility rate
UNDP: United Nations Development Program
