# Supplementary material for: Composite measures of women’s empowerment and their association with maternal mortality in low-income countries
Source: BMC Pregnancy Childbirth. 2017 Nov 8;17(Suppl 2):337. doi: 10.1186/s12884-017-1492-4 (PMC5688512; doi:10.1186/s12884-017-1492-4)
Supplement: Additional file 1: — Open peer review. (PDF 83 kb) [file 12884_2017_1492_MOESM1_ESM.pdf]

Reviewer reports

Title: Composite measures of women's empowerment and their association with maternal mortality in low-income countries

Reviewer 1 : Kavita Singh Ongechi

Major Revisions

1. Abstract: Results: Add in coefficients and p values for the results presented.
2. Abstract: Conclusion: The conclusion should focus on implications of the findings. As it is currently written, the conclusion repeats the results section.
3. Introduction: In terms of reference 9, was quality of services discussed in the article?
4. Introduction: line 27: Expand upon the statement starting on line 24. Explain the mechanism of how women's status could affect MMR.
5. General: There is a lot of use "appear" and "seem" to explain results. Use more definitive words.
6. Introduction: Line 43: Explain what addition analysis is needed.
7. Introduction: Line 56: Have any studies used the Human Development Index? If so, summarize and explain why this might be important. Are measures of poverty and development, which are broader than women's empowerment, also important to look at? Can they be incorporated into the paper?
8. Introduction: Line 83: Explain why transparency/corruption levels are important to maternal mortality? A little background in the introduction would be important.
9. Methods: Corruption Index: Explain perceived corruption by whom?
10. Methods: Explain why GDP was used. Why not also use the Human Development Index? This index might capture a country's infrastructure and poverty levels? What about measures of provider to patient rations, distance or availability of services?
11. Discussion: It would be good to expand upon what perceived corruption is capturing in Africa. Perhaps comment 10 above could be helpful in expanding upon what this means. Is perceived corruption capturing infrastructure levels or availability of services? Or could it partly be trust in the government?
12. Conclusion: Last sentence should be in the discussion and expanded upon there. Provide references indicated that unauthorized user fees are common in Africa.

1. p.5. Paragraph starting with "In the past few decades…" might need some references to support the statements. For example what are the changes in women's political positions etc. What is the evidence that such changes have increased women's status and earning potential. Is this hypothesized or is there actual evidence.

2. P.6. What is the evidence that women leaders bring more funding to maternal health? Expand on references 19 and 20.
3. P.20. How can women take a more active role to address corruption? Expand upon reference 41.

Level of Interest - An article of importance in its field

Quality of written English - Acceptable

I declare that I have no competing interests.

Reviewer 2: Koki Agarwal

Reviewer's report

I enjoyed reading the article. It is an important topic to explore as countries still face persistent challenges to reducing MMR. However, I am not sure why the authors have not included the immediate variables that impact MMR such as SBA, CPR in the analysis?

- Minor Essential Revisions

Discussion on correlation effects of the various Gender Empowerment used. The indicators included in one composite index are acknowledged as correlated with those included in another Index. How is the interaction controlled for in the analysis? How do we explain low income countries like Nepal and Bangladesh that met MDG 5 but probably have very low Gender empowerment score?

Is there any value in attempting to conduct a similar analysis in countries that met MDG 5?

I don't believe the analysis of the corruption score for SS African countries is well explained. Need to expand on the rationale – or suggest further analysis in this area. The linkage to user fee is a bit of a stretch. Many countries have eliminated user fee but women end up paying for services because commodities are not available, I am not sure that would amount to corruption

Policy implications of this study need to be strengthened. Not sure what the take away would be for a MOH official in a low income country with high level of gender disparity

Level of interest - An article of importance in its field
Quality of written English – Acceptable

I declare that I have no competing interests

Response to reviewers

Reviewer 1's Comments

Major Revisions

1. Abstract: Results: Add in coefficients and p values for the results presented.

   Response: Coefficient and p values were added for the results presented in the abstract.

2. Abstract: Conclusion: The conclusion should focus on implications of the findings. As it is currently written, the conclusion repeats the results section.

   Response: We have removed the sentences that repeated the results section and added the implications of the findings in the conclusion.

3. Introduction: In terms of reference 9, was quality of services discussed in the article?

   Response: The authors mentioned that other elements of are and quality of service might have a strong role in survival of severe maternal morbidity (p. 1754) and stated that "if substantial reductions in maternal morality are to be achieved, universal coverage of life-saving interventions needs to be matched with comprehensive emergency care and overall improvements in the quality of maternal health care" (p. 1755).

4. Introduction: line 27: Expand upon the statement starting on line 24. Explain the mechanism of how women's status could affect MMR.

   Response

5. General: There is a lot of use "appear" and "seem" to explain results. Use more definitive words.

   Response: replace the words

6. Introduction: Line 43: Explain what addition analysis is needed.

   Response

7. Introduction: Line 56: Have any studies used the Human Development Index? If so, summarize and explain why this might be important. Are measures of poverty and

development, which are broader than women's empowerment, also important to look at? Can they be incorporated into the paper?

Response:

8. Introduction: Line 83: Explain why transparency/corruption levels are important to maternal mortality? A little background in the introduction would be important.
   Response: add background information to explain the importance of transparency to maternal mortality. [INTRODUCTION]

9. Methods: Corruption Index: Explain perceived corruption by whom?
   Response: Corruption Index is defined as the perceived level of public sector corruption by the respondent. The information was added to clarify the measurement (see page 14).

10. Methods: Explain why GDP was used. Why not also use the Human Development Index? This index might capture a country's infrastructure and poverty levels? What about measures of provider to patient rations, distance or availability of services?

    Response: We thank the Reviewer for these suggestions and agree that measures of provider to patient rations, distance, or availability of services would be informative; however, most countries did not provide these data and we were unable to include those data in this current study. We did not use Human Development Index in this study because the measurement itself captures XX. We have included these suggestions for future studies in the discussion section (see page xx).

11. Discussion: It would be good to expand upon what perceived corruption is capturing in Africa. Perhaps comment 10 above could be helpful in expanding upon what this means. Is perceived corruption capturing infrastructure levels or availability of services? Or could it partly be trust in the government?

    Response:

12. Conclusion: Last sentence should be in the discussion and expanded upon there. Provide references indicated that unauthorized user fees are common in Africa.

    Response: We appreciate the Reviewer's suggestion and have moved the last sentence in the conclusion into discussion (see page 28 line 346). Additionally, we have expanded the discussion with references.

1. p.5. Paragraph starting with "In the past few decades…" might need some references to support the statements. For example, what are the changes in women's political positions etc. What is the evidence that such changes have increased women's status and earning potential. Is this hypothesized or is there actual evidence.

Response: We have added four references to support the statements that women's status has indeed improved significantly. Specifically, reports from UNICEF, UN, World Bank, as well as the Inter-Parliamentary Union were added (please see page 5 for details).

2. P.6. What is the evidence that women leaders bring more funding to maternal health? Expand on references 19 and 20.

Response: We have now added specific evidence that when there are more female policy-makers or parliamentarians, more funding goes to health and social welfare programming. . The following sentences have been added: "For instance, a study in India found that for every one standard deviation increase in the number of female political representatives, a 1.5% reduction in neonatal mortality occurred, because women politicians were more likely to support antenatal care and health facilities [25]. Similarly, in Rwanda, the rise of women parliamentarians led to a major increase in national budget expenditures on healthcare: from 3% in 1998 to 12% in 2006 [26]. This has been directly attributed to women parliamentarians' lobbying activities [26]. After countries introduce gender quotas that increase political participation by women, they tend to spend 3.4 percentage points more on social welfare than do countries without them [24]." (please see pages 6-7).

3. P.20. How can women take a more active role to address corruption? Expand upon reference 41.

Response: We have expanded this section and include examples of how women have taken a more active role in addressing corruption. Specifically, the following sentences were added: "One strategy is to increase women's participation in healthcare governance and in monitoring service delivery. Another strategy is to incorporate women's organizations, such as associations of women journalists, into mobilizing public action against corrupt practices. For example, women's organizations in the Philippines have been at the forefront of anti-corruption efforts, including exposing a high-level criminal network that was trafficking in women and misusing public funds [59]. In general, women seem more sensitive to corruption and less corruptible when they are in positions of power [59]. Hence, efforts to achieve gender equality in the political and management spheres are also considered anti-corruption strategies." (please see page 21).

Reviewer 2's comments:

Reviewer's report

I enjoyed reading the article. It is an important topic to explore as countries still face persistent challenges to reducing MMR. However, I am not sure why the authors have not included the immediate variables that impact MMR such as SBA, CPR in the analysis?

> **Response:** We agree with the Reviewer that most obstetric complications could be managed or prevented if there were skilled birth attendants (SBA, such as doctor, nurse, midwife) women in labor had access to. However, despite the improved coverage of SBA globally, about half of the births in African region and south-east Asian region were not attended by SBA. Since many low and middle income countries did not report on SBA, we were not able to include SBA in the analysis of this current study.

- Minor Essential Revisions

Discussion on correlation effects of the various Gender Empowerment used. The indicators included in one composite index are acknowledged as correlated with those included in another Index. How is the interaction controlled for in the analysis? How do we explain low income countries like Nepal and Bangladesh that met MDG 5 but probably have very low Gender empowerment score?

> **Response:** interaction was not controlled for. Expand discussion and provide hypothesis on low income countries met MDG 5 but with low gender empowerment score.

Is there any value in attempting to conduct a similar analysis in countries that met MDG 5?

> **Response:**

I don't believe the analysis of the corruption score for SS African countries is well explained. Need to expand on the rationale – or suggest further analysis in this area. The linkage to user fee is a bit of a stretch. Many countries have eliminated user fee but women end up paying for services because commodities are not available, I am not sure that would amount to corruption

Response: Expand on the rationale. Future analysis is needed in this area. Expand on the alternative explanation of user fee.

Policy implications of this study need to be strengthened. Not sure what the take away would be for a MOH official in a low income country with high level of gender disparity

> **Response:** expand on policy implications
